# Editorial: Autophagy modulation in cancer treatment utilizing nanomaterials and nanocarriers

**DOI:** 10.3389/fonc.2023.1217401

**Published:** 2023-06-06

**Authors:** Marco Cordani, Maria Condello, Stefania Meschini, Raffaele Strippoli

**Affiliations:** ^1^ Department of Biochemistry and Molecular Biology, School of Biology, Complutense University, Madrid, Spain; ^2^ Instituto de Investigaciones Sanitarias San Carlos (IdISSC), Madrid, Spain; ^3^ National Center for Drug Research and Evaluation, National Institute of Health, Rome, Italy; ^4^ Department of Molecular Medicine, Sapienza University of Rome, Rome, Italy; ^5^ National Institute for Infectious Diseases L. Spallanzani IRCCS, Rome, Italy

**Keywords:** nanomaterials, nanocarriers, drug delivery, cancer therapy, autophagy

Autophagy is a cellular self-degradation process which plays an important role in cellular homeostasis by eliminating malfunctioning proteins and damaged organelles favoring cellular regeneration (1). In cancers, this process may maintain genetic stability and favor cell survival promoting drug resistance. However, in some contexts, autophagy can also induce tumor suppressor mechanisms by activating cell death after exposure to several environmental stresses, including treatment with anti-cancer agents (2). Thus, since autophagy has a dual role in cancer, its pharmacological modulation can, depending by tumor tissue, stage, and metabolic/environmental context, either suppress or promote cancer cell survival.

Recent advances in the modern field of nanotechnology make possible to counteract human diseases with effective bioactive compounds, overcoming the obstacles of traditional drugs such as biodistribution, biocompatibility and degradation or stability (3). Interestingly, nanomaterials have been explored as potent modulators of autophagy through multiple mechanisms and have been exploited as therapeutic agents against cancer (4). The relevance of nanomedicine for autophagy modulation is remarked by several clinical trials that have been set up in the last years (5). This suggests that the application of encapsulated drugs active in autophagy is effective in clinical practice and it would acquire more clinical relevance in the near future, as a complementary therapy for the treatment of cancers.

This Research Topic gathers original research and review papers on novel drugs in cancer treatments based on autophagy modulation as well as the application of novel nanomedicines capable of modulating autophagy, and/or new discoveries in autophagy-related signaling pathways in cancer. The 9 accepted articles consist of 5 Original Research articles, 3 Reviews and 1 Brief Research article. Overall, these studies demonstrate that inhibition or enhancement of the autophagy pathway may serve as an effective tool to counteract cancer.

In the last years, many herbal medicines and bioactive natural products have been explored for their antitumoral efficacy through modulation of key cellular signaling routes involving autophagy (6). In this regard, the Review Article by Wang et al. and Chavda et al. focused on the therapeutic effects and mechanisms of plant-derived natural products as well as combination therapy on cancer disease. The authors discussed the tumor suppressor role of several natural compounds in autophagy modulation and the recent strategies of their encapsulation to generate effective nano-delivery tools for targeted therapies in cancer. The translational potential of such nanoformulations in the clinic is also discussed. This review aims to elicit the interest of the community for developing anticancer strategies in both cellular and animal models, with high efficacy and low side effects.

Nazir et al. described the synthesis of 3-O-prenyl glycyrrhetinic acid (NPC-402), a derivative of glycyrrhetinic acid, and reported its cytotoxic activity in melanoma in *in vitro* and *in vivo* models. They showed that NPC-402 induces endoplasmic reticulum stress-mediated autophagy through modulation of an ERK/AKT signaling pathway in melanoma cells and reduces the tumor size and weight without any toxic side effects in C57BL/6J mice. Since methods to encapsulate NPC-402 in nanoformulations have been previously described (7), NCP-402 has the potential to become a nanomedicine for autophagy modulation for melanoma treatment. Hepatocellular carcinoma (HCC) is the third most common cause of cancer-related death globally (8). Despite many efforts made to find effective therapeutic strategies, HCC often becomes resistant to Sorafenib, the first-line chemotherapy, and only 15% of liver cancer patients survive at 5 years from diagnosis. In a study led by Sun et al. the effect of 5-chloro-N′-(2,4-dimethoxybenzylidene)-1H-indole-2-carbohydrazide (IHZ-1/ZJQ-24), a novel indole hydrazide derivative with bioactive proprieties for the management of liver cancer, has been evaluated. The authors showed that treatment with IHZ-1 in HCC cell lines increases the generation of intracellular reactive oxygen species (ROS) and induces autophagy through the activation of a ROS/JNK pathway. Interestingly, an indole hydrazide derivative has been encapsulated in different types of nanomaterials, such as hydrogels, pH-controlled biopolymers, or metal oxide nanoparticles, for their efficient delivery in cancer cells (9–11). Paclitaxel (PTX) and norcantharidin (NCTD) are anticancer compounds that have been described to be active in autophagy mechanisms (12–14). Xie et al. have generated PTX/NCTD-loaded core–shell lipid nanoparticles modified with a tumor neovasculature-targeted peptide, APRPG and investigated their anti-tumor effects in HCC. These functionalized nanostructures were reported to alter a complex network of signaling pathways involved in the migration and proliferation of liver cancer cells and reduced the volume and growth of HCC in animal models without side effects in healthy tissues. The antitumor activity of zinc-oxide nanospheres (ZnO-NS) has been explored for liver cancer treatment. In this regard, Hassan et al. showed that ZnO-NS synthesized by the Sol-gel method induced strong cytotoxic stress, ROS accumulation and lipid peroxidation in HuH7 cell line. They also reported lipid droplets accumulation and alterations in mitochondria leading to cell death. On the other side, the biochemical and mechanical proprieties of tumor microenvironment may affect drug distribution and release at the tumor site. To overcome this barrier, Yeow et al. produced a recombinant fusion protein consisting in tumor necrosis factor-α (TNF-α) and CSG peptide (CSGRRSSKC) to deplete extracellular matrix in liver cancer generated in mice models. This treatment was able to enhance an intra-tumoral accumulation of iron-oxide nanoparticles (IO-NPs) as determined by magnetic resonance imaging analysis. Since IO-NPs have been extensively studied as autophagy modulators in cancer therapy (15, 16) this study may set the basis for combined therapies based on the modulation of tumor microenvironment in combination with metal-oxide nanostructures to intervene on cancer autophagy.

Breast cancer is the most common cancer that affect women worldwide. Despite significant progress, breast cancer remains the tumor with the highest mortality. Autophagy plays an important role during breast cancer genesis and progression participating in many phenotypic traits such as migration, invasion, and therapy resistance (17). Nanomedicine is a promising strategy for breast cancer treatment. Products such as Doxil^®^ and Abraxane^®^ have already been used for breast cancer treatment as adjuvant therapy with favorable clinical outcomes (18). The review article authored by Gharoonpour et al. summarizes the advantages and disadvantages of nanoparticles-based therapy in breast cancer through autophagy modulation. The authors thoroughly discuss the translational potential of several nanoformulations combined with traditional chemotherapy, and the different phases of clinical trials involving nanomedicine that have been set up for breast cancer treatment. Moreover, Zhang et al. explored the therapeutic potential of chitosan/alginate nanoparticles for co-delivery of anti-autophagy drugs doxorubicin and hydroxychloroquine in breast cancer cells. This nanodelivery system was able to efficiently encapsulate these anti-autophagy drugs and allow their pH-sensitive release in the tumoral site.

Hence, the importance of the knowledge of the regulatory mechanisms of autophagy, along with the potential opportunities presented by newly emerging nanocarriers and nanomaterials suitable for bypassing developed cellular resistance, makes this topic particularly interesting. We believe that these advances will inspire basic and clinical scientists working in related fields to further investigate molecular mechanisms and to perform translational studies.

## Author contributions

MCor wrote the original draft and revised the manuscript. The other authors revised the manuscript and provided valuable suggestions. All authors listed have approved this manuscript for publication.
